# Chemical compositions and antifungal activities of *Satureja macrosiphon* against *Candida* and *Aspergillus* species 

**DOI:** 10.18502/cmm.5.4.2162

**Published:** 2019

**Authors:** Marjan Motamedi, Mohammad Jamal Saharkhiz, Keyvan Pakshir, Sara Amini Akbarabadi, Marzieh Alikhani Khordshami, Fatemeh Asadian, Zahra Zareshahrabadi, Kamiar Zomorodian

**Affiliations:** 1 Department of Medical Parasitology and Mycology, School of Medicine, Shiraz University of Medical Sciences, Shiraz, Iran; 2 Department of Horticultural Sciences, Faculty of Agriculture, Shiraz University, Shiraz, Iran; 3 Basic Sciences in Infectious Diseases Research Center, Shiraz University of Medical Sciences, Shiraz, Iran; 4 Department of Pathology, School of Medicine, Shiraz University of Medical Sciences, Shiraz, Iran; 5 Department of Medical Parasitology and Mycology, School of Public Health, Tehran University of Medical Sciences, Tehran, Iran

**Keywords:** Essential oil, MTT assay, Satureja macrosiphon, XTT assay

## Abstract

**Background and Purpose:**

Despite the various applications of *Satureja* species, there are limited data in this domain. Regarding this, the present study was conducted to investigate the essential oil (EO) biological activity of *S. macrosiphon* species in Iran.

**Materials and Methods:**

The EO of *S. macrosiphon* flowers was obtained by hydrodistillation. Chemical compositions of the EO were analyzed using gas chromatography-mass spectrometry. In addition, minimum inhibitory concentrations (MIC) were measured by means of the broth microdilution method. The estimation of antibiofilm and cytotoxic activities was also accomplished using the tetrazolium salt and MTT assays, respectively.

**Results:**

A total of 26 components were identified in the EO with linalool as the main constituent (28.46%). A MIC range value of 0.25-8 μL/mL was obtained against all of the tested fungi. The EO inhibited the biofilm development of the *Candida* tested strains at a concentration of 4-8 μL/mL. Cytotoxicity (IC_50_) of EO against the HeLa cell was greater than the MIC concentration (6.49 μL/mL).

**Conclusion:**

Based on the findings, it was concluded that the EO of *S. macrosiphon* has the potential for further use as an antifungal agent.

## Introduction

During the past two decades, there has been a growth in the population of the immunocompromised patients vulnerable to invasive fungal infections. This rise is mostly due to the adoption of novel treatments, such as hematopoietic stem cell and solid organ transplantation, coupled with the use of newer and more potent chemotherapeutic and immunomodulatory agents (1-4). Among different species, *Candida* and *Aspergillus* are the most prevalent fungal pathogenic agents responsible for the majority of the infections (5). 

*Candida* species are considered as the main cause of nosocomial fungal infections (almost 80%) (6). Although *C. albicans* are still responsible for the majority of the candidiasis cases and rank seventh among all hospital pathogens, the number of infections caused by non-*albicans Candida* species is also increasing (7). Accordingly, almost 50% of bloodstream infections in surgical and neonatal intensive care units are caused by non-*albicans Candida* (8). In addition, *Aspergillus* species is responsible for 1.3% of fungal nosocomial infections (9). The incidence of *Aspergillus *infection appears to be much higher in special care wards, such as bone marrow transplant units (10). More than 95% of aspergillosis cases are caused by *A. fumigatus* and *A. flavus *(11). 

The emergence of resistance in these saprophytic fungi responsible for invasive fungal infections has complicated their treatment (12). In this regard, there are several reports describing in vitro and clinical resistance to azole and echinocandins among *Candida* and *Aspergillus* species (13-15). In the face of increasing resistance to antifungal drugs, natural products and phytochemicals are being widely screened as the potential sources of novel antifungal agents (16-18). Traditionally, plants have been used to prevent and cure infectious diseases. As part of plant defense mechanisms against microbial pathogens, they produce aromatic chemicals and secondary metabolites. Among these secondary metabolites, essential oils (EOs) obtained by the hydrodistillation of the plants are known to have antimicrobial activities. 

*Satureja *is a genus of the well-known medicinal plant of *Lamiaceae* family and comprises numerous species growing wild in the Mediterranean region (19). The EO isolated from various species of *Satureja* has been shown to have biological and pharmacological activities, such as antibacterial (20), fungicidal (21), antiviral (22), and antioxidant (23). The genus *Satureja* embraces over 30 species growing in the eastern parts of Mediterranean region. Fourteen species of this genus are growing wild in the western, central, and northern parts of Iran, among which *S. macrosiphon* is one of the endemic species in this country. The present study was conducted to determine the chemical constituents of *S. macrosiphon* EO. This study also involved the investigation of the antifungal, antibiofilm, and cytotoxic activities of this EO against the different species of *Candida* and *Aspergillus*.

## Materials and Methods

***Plant material and essential oil preparation***

Flowers of species were used for the analysis of EO composition. To this end, 50 g of dried flowers was subjected to hydrodistillation for 3 h using a Clevenger-type apparatus to produce oil. The obtained EO was dried over anhydrous sodium sulfate and stored in sealed vials at low temperature (4°C) before analysis.

***Essential oil analysis and identification procedure***

The chemical composition of EO was determined by gas chromatography-mass spectrometry (GC-MS) using a CP-SIL 5HP fused silica column. The GC (Trace GC ULTRA, Thermo Fischer) analysis was performed using a flame ionization detector (GCFID) with a Varian capillary column (CP-SIL 5HP, length of 60 m, diameter of 0.25 mm, and film thickness of 0.25 μm). The column temperature was programmed at 60-280°C for 4°C/min. Furthermore, the temperature of the injector was fixed at 250°C. Helium was used as the carrier gas at a flow rate of 1.1 mL/min and a split ratio of 1/50. The quadrupole mass spectrometer was scanned over 35-465 amu with an ionizing voltage of 70 eV and an ionization current of 150 mA. The GCFID analysis of the oil was conducted using a Thermoquest-Finnigan instrument equipped with a DB-5 fused silica column (60 m*×*0.25 mm, film thickness of 0.25 mm). 

Nitrogen was used as the carrier gas at a constant flow of 1.1 mL/min, and the split ratio was the same as that used for GC-MS. The oven temperature was raised from 60°C to 250°C at a rate of 4°C/min and held for 10 min. The injector and detector (FID) temperatures were kept at 250°C and 280°C, respectively. Semi-quantitative data were obtained from FID area percentages without the use of correction factors. Retention indexes (RIs) were calculated using the retention times of n-alkanes (C_6_-C_24_) that were injected at the same temperature and conditions. The compounds were identified by comparing their RI with those reported in the literature, and their mass spectrum was compared with those reported in the Wiley Library (24).

***Fungal strain***

The antifungal activities of the EO were determined against 25 standard fungal strains. These strains included *C. albicans *(ATCC 5982, 1912, 562, 1905, 1949, 10261, and 2730), *C. tropicalis *(ATCC 750), *C. krusei *(ATCC 6258), *C. glabrata *(ATCC 863, 2192, 2175, 6144, and 90030), *C. dubliniensis *(CBS 8501, ATCC 8500, 7987, and 7988), *C. parapsilosis *(ATCC 4344), *C. neoformance *(ATCC 9011), *Aspergillus flavus *(ATCC 64025), *A. fumigatus *(ATCC 14110, CBS 144.89), *A. clavatus *(CBS 514.65), and* A. oryzae *(CBS 818.72). Moreover, 35 clinical strains that were previously isolated from the oral or vaginal infections and identified by polymerase chain reaction-restriction fragment length polymorphism were used in this study. 

***Determination of minimum fungicidal concentration and minimum fungicidal concentration***

The MIC of EO was determined according to broth microdilution assay as recommended by the clinical and laboratory standards institute (CLSI) for yeasts (M27-A3) and molds (M38-A2) (25), with some modifications. In this regard, a range of EO concentrations (0.06-16 μL/mL) was prepared in 96-well microtiter plates (Nunc) using RPMI-1640 media (Sigma, St. Louis, MO, USA) buffered with MOPS (Sigma, St. Louis, MO, USA). The inocula of the yeasts were prepared from 24-hour solid cultures, and suspensions were adjusted to 0.5 McFarland standard turbidity at a wavelength of 630 nm (yielding a stock suspension of 1-5*×*10^6^ cells/mL). For *Aspergillus *species, conidia were recovered from the 7-day-old cultures grown on potato dextrose agar by a wetting loop with Tween 20. The collected conidia were transferred in sterile saline, and their turbidity was adjusted to the optical density of 0.09-0.11 yielding 0.4-5*×*10^6^ conidia/mL. 

The working suspension was prepared by making 1/50 and 1/1000 dilutions with the RPMI of the stock suspension for molds and yeasts, respectively. After the addition of 0.1 mL of the inocula to the wells, the trays were incubated at 30°C for 24-48 h in a humid atmosphere. In addition, 200 *μ*L of the uninoculated medium was included as a sterility control (blank). Growth controls (medium with inoculums and 5% [v/v] without the EO) were also included. 

The growth in each well was compared with that of the control well. The MICs were visually determined and defined as the lowest concentration of the EO that produced no visible growth. To this end, 10 *μ*L of culture was taken from each well where growth was not observed on the SDA plates and then incubated at 30°C for 48 h. The minimum fungicidal concentration (MFCs) was determined as the lowest concentration yielding no more than four colonies, which corresponds to fungal mortality of 98% in the initial inoculums. Fluconazole was used as a positive control in each experiment.

***Inhibition of Biofilm Formation***

Serial dilution of the EO (0.015-8 μL/mL) was prepared in RPMI-1640 in 96-well microtiter plates. After the addition of 0.1 mL of the suspension of *C. albicans* and *C. dubliniensis* (5×10^6^ cells/mL) inocula to the wells, the trays were incubated at 30°C for 24-48 h in a humid atmosphere. In addition, 200 *μ*L of the uninoculated medium was included as a negative control (blank), while RPMI with yeasts but without EO served as a positive control. Metabolic activity was assessed using the tetrazolium salt (XTT) assay (26). Briefly, the wells coated with *Candida* biofilms were washed with phosphate-buffered saline (PBS). Then, 0.5 mg/mL XTT (Sigma) and 10 mM menadione (Sigma) were added in 100 μL of PBS. The plates were incubated in dark for 2 h at 37°C and then gently agitated. The XTT formazan was measured colorimetrically at 490 nm.

***Cytotoxicity evaluation***

HeLa cell was obtained from the National Cell Bank of Iran, Pasteur Institute, Tehran, Iran. The cell line was maintained in RPMI-1640 supplemented with 10% fetal bovine serum, 100 units ⁄ mL penicillin-G, and 100 μg ⁄ mL streptomycin. The cell was grown in monolayer culture at 37°C in humidified air containing 5% CO_2_. Cell viability following exposure to EO was estimated using the MTT reduction assay (27). HeLa cells were plated at the densities of 1×10^5^ and 2.5×10^4^ cells ⁄ mL. The control well contained no EO, and blank wells contained only growth medium for background correction. After overnight incubation at 37°C, half of the growth medium was removed, and 50 μL of the medium, supplemented with different concentrations (0.03 to 16 *μ*L/mL) of EO dissolved in RPMI-1640, was added. The cells were incubated for 72 h. At the end of the incubation time, the medium was removed, and MTT was added to each well at a final concentration of 0.5 mg ⁄ mL. Subsequently, the plates were incubated for another 4 h at 37°C. In the next stage, formazan crystals were solubilized in 200 μL DMSO. The optical density was measured at 570 nm with background correction at 655 nm using a Bio-Rad microplate reader. Doxorubicin was used as a positive control against HeLa cells in the same setting. The percentage of viability inhibition compared to control wells was calculated for each concentration of the compound, and IC_50_ values were calculated using the CURVEEXPERT software, version 1.34 for Windows (Hyams Development, OH, USA).

## Results

The identified chemical components of the EO obtained from the aerial parts of *S. macrosiphon *are presented in Table 1. A total of 26 components were identified, representing 98.70% of the total EO. The major constituents of the EO were linalool (28.46%), borneol (16.22%), and terpinene (14.58%). The antifungal activities of *S. macrosiphon* EO against the examined fungi are demonstrated in Table 2. The results showed that the EO inhibited the growth of all standard *Candida* species at concentrations of 0.25-4 μL/mL. Furthermore, the EO exhibited fungicidal activity (MFC) against the tested yeasts at a concentration range of 0.5-8 μL/mL. Moreover, the MIC and MFC values for the standard species of *Aspergillus*, which were sensitive to the EO, were in the range of 0.25 to 2 μL/mL and 0.5 to 4 µl/ml, respectively (Table 2).

As indicated in Table 3, the EO of* S. macrosiphone* showed effective antibiofilm activity against tested *Candida* species and inhibited the biofilm formation of *C. albicans* and *C. dubliniensis* at the concentrations of 4 and 8 μL/mL, respectively. Moreover, the cytotoxic activity of the EO in HeLa cells was evaluated and showed an IC_50 _value of 6.49 μL/mL.

**Table 1:** Chemical composition of the essential oil of *Satureja macrosiphon* growing in Iran

**Components**	**RI**	**Composition (%)**
Linalool	1096	28.46
Borneol	1169	16.22
Terpinene-4-01	1177	14.58
Cis-sabinene hydrate	1070	12.96
-terpinene	1059	4.21
Camphene	945	3.22
-terpinene	1017	2.40
Caryophyllene oxide	1583	1.86
- pinene	939	1.82
-terpineol	1188	1.60
(z)-caryophyllene	1408	1.49
Bornyl acetate	1288	1.31
Geranyl acetate	1381	1.26
P-cymene	1024	1.17
Cis-p-menth-2-en-1-ol	1121	0.84
Terpinolene	1088	0.80
Sylvestrene	1030	0.77
Camphor	1146	0.69
Germacrene D	1485	0.64
-pinene	979	0.60
Bicyclogermacrene	1500	0.51
Trans-linalool oxide	1086	0.50
Sabinene	975	0.41
-thujene	930	0.39
Total		98.71

RI: retention index

Compounds listed in order of elution from a DB-5 column.

## Discussion

The species of genus *Satureja* have known aromatic EOs with medicinal properties (28). Among different species of this plant, *S. macrosiphon* is a rare species growing wild in Iran. In this study, the main compound of *S. macrosiphone* EO was determined as linalool (28.46%), while Amiri et al. reported alpha-terpineol (26.7%) as the main constituent of the EO of this species (29). Similar to our study, they found borneol as the second most frequent compound of EO (29). The difference between the chemical composition of the constituents might be due to the different genotype, geographical location, or environmental conditions of the plant materials. 

**Table 2:** Antifungal activity of *Satureja macrosiphon* extracts against the fungal strains tested based on broth microdilution assay

**Tested ** ***Candida*** ** and ** ***Aspergillus*** ** spp. isolate**	**Number of isolate**	**MIC (μL/mL)** **GM** ^a^ ** (range)**	**MFC (μL/mL)** **GM** ^a^ ** (range)**
*Candida *spp. Standard isolate	*C. albicans *(6)	1.5 (1-2)	2.6 (2-4)
	*C. dubliniensis *(5)	2.2 (1-4)	3.6 (2-4)
	*C. glabrata *(5)	0.7 (0.5-1)	1.4 (1-2)
	*C. tropicalis *(1)	0.5	1.0
	*C. parapsilosis *(1)	4.0	4.0
	*C. krusei *(1)	0.25	1.0
*Candida* spp. azole-sensitive clinical isolates	*C. albicans *(9)	1.5(0.06-2)	2.7(1-4)
	*C. dubliniensis *(3)	1.1(0.5-2)	2.3(1-4)
	*C. glabrata *(1)	0.12	4.0
	*C. tropicalis *(2)	1.5(1-2)	1.5(1-2)
	*C. parapsilosis *(3)	0.5(0.25-1)	2.0
*Candida* spp. azole-resistant clinical isolates	*C. albicans *(12)	1.3(0.5-4)	1.9(0.5-4)
	*C. dubliniensis *(2*)*	0.25	2.0
	*C. tropicalis *(3)	2.5(1-4)	3.0(2-4)
*Aspergillus* spp. standard isolate	*A. flavus *(1 )	2.0	4.0
	*A. fumigatus *(2 )	1.1 (0.25-2.0)	2.5 (1-4)
	*A. oryzae *(1 )	2.0	4.0
	*A. clavatus *(1 )	0.25	0.5

GM: geometric mean, MIC: minimum inhibitory concentration, MFC: minimum fungicidal concentration

**Table 3:** Antibiofilm activity of the essential oil of *Satureja macrosiphon* against biofilm formation caused by *Candida albicans* and *Candida dubliniensis*

**Concentration µl/ml**	***C. albicans***		***C. dubliniensis***	
	**Optical density**	**%Viability**	**Optical density**	**%Viability**
8	0.045	0.0	0.047	0.0
4	0.046	0.0	0.053	2.6
2	0.048	0.24	0.127	34.7
1	0.102	6.7	0.172	54.3
0.5	0.220	20.0	0.189	61.7
0.25	0.260	25.0	0.198	65.6
0.12	0.391	41.4	0.206	69.1
0.06	0.426	45.0	0.216	70.0
0.03	0.516	56.0	0.220	75.2
0.015	0.726	81.2	0.241	83.9

It has been shown previously that EOs exhibit their antimicrobial activity by disrupting the cell membrane of microorganisms, inhibiting their enzyme activity, and down-regulating regulatory genes (30). Previous studies demonstrated that the EOs of *Satureja* species are among the most potent EOs with regard to antifungal properties (31, 32). The in vitro antifungal activity of *S. macrosiphone* EO against the examined fungi was quantitatively determined by the CLSI method. According to the results, the EO exhibited great in vitro antifungal activities against all tested fungi at the concentration of up to 4 μL/mL. These results support the data previously reported by Gorran et al. (33), confirming the inhibitory activity of *S. macrosiphone* EO against the growth and mycotoxin production of *A. flavus*. 

According to the literature, there is a relationship between the major component of the EO and its antimicrobial activity. Similar antifungal activity was also reported for the EO of *S. montana*, with linalool as the dominant component (34, 35). It has been reported that linaloo, as terpene alcohol, has considerable antimicrobial activity against a large number of microorganisms (36-38). Trombetta et al. showed that linaloo might protuberate the microbial plasma membrane, which in turn results in the alteration of cellular permeability and leakage of intracellular organelles and ions (30). In addition, the EO inhibited the azole-resistant strains and even exhibited fungicidal activities against all examined fungi at the concentrations of up to 4 µl/ml. No significant differences were found between azole-resistant and -susceptible clinical strains in terms of inhibitory concentration.

In recent decades, fungal biofilms have become of greater significance in the clinical context since these communities are associated with drastically enhanced ability to express resistance against most antifungal agents (39). In this regard, *Candida* species are the most famous fungi responsible for biofilm formation. In the present study, the formation of biofilm was inhibited completely at a concentration of up to 2 μL/mL in a dose-dependent manner. This finding is similar to that of the study performed by Manoharan et al. who reported on the strong antibiofilm activity of linalool against *C. albicans* (40). To the best of our knowledge, the cytotoxic activity of *S. macrosiphone* EO has not been reported yet. The cytotoxicity (IC_50_) of the EO against the HeLa cells was greater than its MICs and MFCs against the examined fungi. Therefore, this product might be used safely for pharmaceutical applications. However, it is required to perform further studies on other cell lines and carry out experimental studies on animals to accurately assess the cytotoxicity of this EO.

## Conclusion

The findings of the present study were indicative of antifungal, antibiofilm, and cytotoxicity activities of *S. macrosiphone* against pathogenic fungi. In addition, the data provided support to use this EO in the production of antifungal agents and pharmaceutical products. As all of the tests were performed in vitro, further studies are still needed to see if a fungal infection can be treated by *S. macrosiphone* EO. 
